# Free heme induces neuroinflammation and cognitive impairment by microglial activation via the TLR4/MyD88/NF-κB signaling pathway

**DOI:** 10.1186/s12964-023-01387-8

**Published:** 2024-01-05

**Authors:** Xin Wei, Fan Zhang, Dan Cheng, Zhongyu Wang, Na Xing, Jingjing Yuan, Wei Zhang, Fei Xing

**Affiliations:** 1 Department of Anesthesiology, Pain and Perioperative Medicine, The First Affiliated Hospital of Zhengzhou University, Zhengzhou, 450052 China; 2 Henan Province International Joint Laboratory of Pain, Cognition and Emotion, Zhengzhou, 450052 China

**Keywords:** Free heme, Cognitive function, Neuroinflammation, Neuron, Microglia activation, TLR4/MyD88/NF-κB

## Abstract

**Background:**

Red blood cells (RBCs) transfusion is related to perioperative neurocognitive disorders. The toxic effect of free heme has been identified in many pathologies. However, the underlying mechanisms of RBCs transfusion or free heme in cognitive impairment have not been clearly explored. Therefore, this research was conducted to determine the mechanism of free heme-induced neuroinflammation and cognitive impairment.

**Methods:**

Rats were received intraperitoneal injection of hemin alone or combined with intracerebroventricular injection of Hemopexin (HPX), and MWM test was conducted to measure cognitive function. The amount of heme-HPX complexes was evaluated by flow cytometry for CD91 + cells. The microglial inflammatory response in rat brain was observed by immunofluorescence staining of Iba-1, and the inflammatory factors of TNF-α, IL-1β and IL-6 in rat brain and BV2 cells were detected by ELISA analysis. Furthermore, neuronal apoptosis in HT22 cells alone and in HT22 + BV2 coculture system was detected by flow cytometry and immunofluorescence staining. Finally, western blot was conducted to detect TLR4/MyD88/NF-κB proteins in rat brain and BV2 cells treated with hemin or combined with pathway inhibitors. Additionally, the M1 surface marker CD86 was observed in BV2 cells to further confirm neuroinflammation.

**Results:**

Intraperitoneal injection of hemin induced cognitive impairment, increase of CD91 + cells, up-regulation of TNF-α and IL-1β, down-regulation of IL-6, activation of microglia, and activation of the TLR4/MyD88/NF-κB signaling pathway in rat brain. Significantly, intracerebroventricular injection of HPX reduced the above effects. Hemin induced boost of TNF-α, IL-1β and IL-6 in BV2 cells, as well as apoptosis in HT22 cells. Notably, when HT22 cells were cocultured with BV2 cells, apoptosis was significantly increased. Hemin also induced activation of the TLR4/MyD88/NF-κB signaling pathway and increased the M1 surface marker CD86 in BV2 cells, and inhibiting this pathway reduced the inflammatory responses.

**Conclusions:**

Free heme induces cognitive impairment, and the underlying mechanism may involve neuronal apoptosis and microglial inflammation via the TLR4/MyD88/NF-κB signaling pathway. HPX may have potential therapeutic effects.

Video Abstract

**Supplementary Information:**

The online version contains supplementary material available at 10.1186/s12964-023-01387-8.

## Background

With more than 100 million units of blood collected worldwide each year for therapeutic purposes, red blood cells (RBCs) transfusion can increase the oxygen-carrying capacity of the blood and is one of the most common treatments for patients [1]. However, RBCs transfusion has significant side effects, including infectious diseases and immunological reactions [2]. Currently, RBCs stored for up to 42 d are approved for transfusion. The storage of RBCs induces modifications of biochemical and biological properties. These ex-vivo alterations are suspected to induce adverse events [3, 4]. Importantly, erythrocytes lysed in the storage bag and ruptured in vivo after transfusion can release free-hemoglobin (Hb) or free-heme, which may be involved in central nervous system (CNS) inflammation and cognitive decline [5, 6]. Clinically, RBCs transfusion, especially when RBCs have been stored for a long time, is related to postoperative delirium (POD) [7, 8]. In rats, transfusion of old RBCs can induce neuroinflammation and cognitive impairment and the effects are mediated by free-Hb [9].

A heme is a ubiquitous iron-containing compound which is made from 4 pyrroles and forms the nonprotein part of Hb and some other biological molecules. It is hydrophobic and readily enters cell membranes [10]. In situations such as RBCs rupture, heme may be released from Hb or other host and in a free state, which is called free heme. The normal level of free heme does not harm the human body, but the accumulation of free heme in excess of metabolic clearance will cause pathological changes [11]. Significantly, for patients who receive RBCs transfusion, the free heme level is dependent on the number of RBCs units transfused [12]. The toxic effect of free heme plays an important role in many pathologies, including not only acute conditions such as intravascular hemolysis but also insidious processes such as neurodegeneration and cardiac dysfunction [13, 14]. Interestingly, when cultured macrophages are exposed to hemolytic aged RBCs, excessive free heme can alter the macrophage phenotype toward a pro-inflammatory state [15].

Perioperative neurocognitive disorders (PND), including POD and postoperative cognitive dysfunction (POCD), are common following anesthesia and surgery in older patients and significantly increase morbidity and mortality [16, 17]. Neuroinflammation in the hippocampus, including microglial inflammatory response and neuronal apoptosis, plays an essential role in cognitive dysfunction [18, 19]. Initial evidence has shown that rats transfused with old RBCs or receiving free-Hb and mice receiving intracerebral heme injection suffer from neuroinflammation and neurological deficits [9, 20]. However, it is still unclear how free heme causes neuroinflammation after RBCs transfusion. Moreover, the heme scavenger hemopexin (HPX) can alleviate cognitive dysfunction in some studies related to intracerebral hemorrhage [21, 22], and heme oxygenase-1 (HO-1), an enzyme that degrades heme, has neuroprotective effects [23].

Toll-like receptor 4 (TLR4) is an important transmembrane receptor involved in inflammatory processes, and free heme can act as a ‘danger’ signal to initiate TLR4 responses [24]. Upon activation, TLR4 can activate different transcription factors through the myeloid differentiation factor 88 (MyD88) pathway, including nuclear factor-kappa B (NF-κB), which eventually induces the production of inflammatory cytokines, such as tumor necrosis factor-alpha (TNF-α), interleukin-1β (IL-1β) and interleukin-6 (IL-6) [25]. There is an ongoing need for exploration of the molecular mechanism of transfusion-mediated neurological impairment. Therefore, in this context, we conducted intraperitoneal injection of hemin (a reagent in the oxidized state of heme and derived from processed RBCs) and intracerebroventricular injection of HPX in rats and observed cognitive function by the Morris water maze (MWM) test. Then, we analyzed the CD91 + cells to evaluate the amount of heme-HPX complexes. The microglial inflammatory response in rat brain was observed by immunofluorescence staining, and the inflammatory factors of TNF-α, IL-1β and IL-6 in rat brain and BV2 cells were detected by ELISA analysis. Additionally, the M1 surface marker CD86 was observed in BV2 cells to further confirm neuroinflammation. Furthermore, neuronal apoptosis in HT22 cells or the coculture system was detected by FCM and immunofluorescence staining. Finally, we observed the expression of the TLR4/MyD88/NF-κB proteins by western blot in rat brain and BV2 cells treated with hemin or hemin + inhibitors to validate the hypothesis that free heme could induce neuroinflammation and cognitive impairment by microglial activation via the TLR4/MyD88/NF-κB signaling pathway.

## Methods

### Animals and experimental protocol

The animal experiments were approved by the Ethics Committee of the First Affiliated Hospital of Zhengzhou University. All animal experiments were carried out in accordance with the National Institutes of Health Guide for the Care and Use of Laboratory Animals. Six-month-old male Sprague–Dawley rats (500-600 g) were obtained from Shanghai Jihui Laboratory Animal Care Co.,Ltd. Animals were maintained under standard controlled environment at constant temperature of 22 ± 2 °C and humidity of 50 ± 10% with 12-h light/dark cycle. Food and water were available ad libitum. The rats were left to acclimatize to the environment for two weeks prior to inclusion in the experiment.

**Fig. 1:**
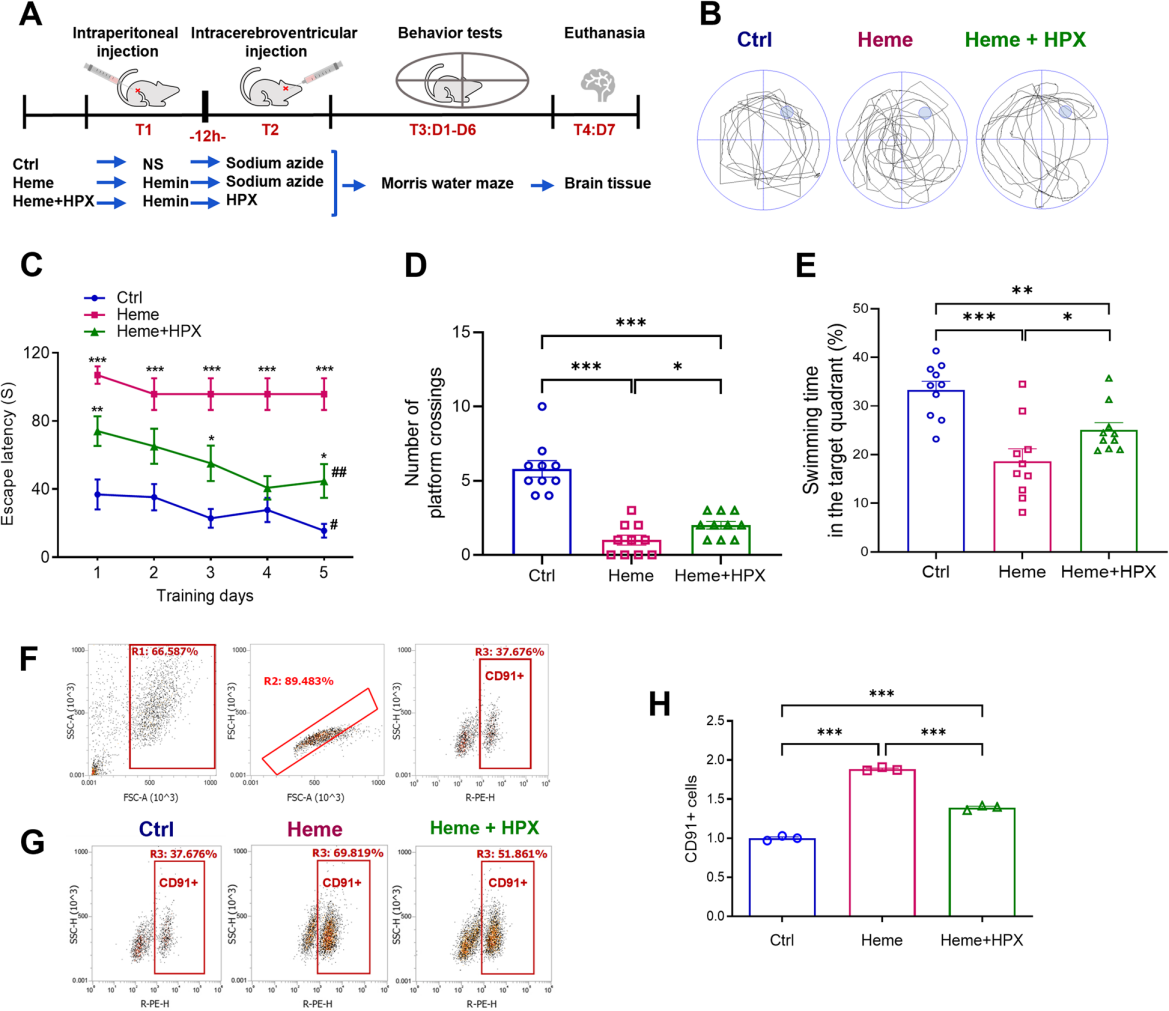
Hemin induced cognitive impairment and increased CD91 + cells in brain, and HPX alleviated these effects.** A** Rats and experimental protocol. The rats were randomized into 3 groups: 1) Control group (*n* = 10); 2) Heme group (*n* = 10); and 3) Heme + HPX group (*n* = 10). On the first day (T1), the control group rats received an intraperitoneal injection of NS. The rats in the Heme group and Heme + HPX group received an intraperitoneal injection of hemin. After 12 h (T2), the control group and Heme group rats received an intracerebroventricular injection of sodium azide, and the Heme + HPX group rats received an intracerebroventricular injection of HPX. Behavior tests were conducted over the next 6 days (T3). Euthanasia was performed on day 7 (T4). **B** Representative swimming paths obtained in the probe trial session of MWM test. **C** The escape latency in the acquisition session for five consecutive days. Data are presented as means ± SEM. ***p < 0.001, **p < 0.01, *p < 0.05 compared to control group. ^##^*p* < 0.01, ^#^*p* < 0.05 compared to the values on day 1. **D** The number of platform crossings in the probe trial session. **E** The swimming time (%) in the target quadrant. **F** The gating strategy of FCM analysis for CD91 + cells in rat brain. Cells were first gated to remove fragments and particles (R1, R2), and then cells expressing CD91 were gated (R3). **G** Representative FACS plots and quantification of the percentage of CD91 + cells in the three groups. **H** Quantification of CD91 + cells among brain cells after normalization. Data are presented as means ± SEM. ***p < 0.001. **p < 0.01. *p < 0.05

The rats were randomized into three groups: 1) Control group (*n* = 10), in which rats received sham operation; 2) Heme group (*n* = 10), in which rats received an intraperitoneal injection of hemin; and 3) Heme + HPX group (*n* = 10), in which rats received an intraperitoneal injection of hemin and an intracerebroventricular injection of HPX. On the first day of the experiment (T1), the control group rats received an intraperitoneal injection of 0.5 mL normal saline (NS). The Heme group and Heme + HPX group rats received an intraperitoneal injection of 0.5 ml hemin (25 mg). After 12 h (T2), the control group and Heme group rats received an intracerebroventricular injection of 5 μl 0.1% sodium azide, and the Heme + HPX group rats received an intracerebroventricular injection of 5 μl (5 μg) HPX. Behavior tests were conducted over the next 6 days (T3). Euthanasia was performed by intraperitoneal injection of pentobarbital sodium (75 mg/kg) on day 7 (T4) after the behavioral tests. The brain tissue of rats was taken, half of which was used for immunofluorescence staining. The other half was made into brain tissue homogenate (Fig. 1A).

### Reagents

Hemin (H9039, Sigma, USA) was freshly dissolved in DMSO and diluted with NS to a final concentration of 50 mg/ml in darkness. Rats in the Heme group and Heme + HPX group were intraperitoneally injected with 0.5 ml (25 mg, 50 mg/kg) hemin. The dose was selected based on previous studies [20, 21, 26–29]. Rats in the control group were intraperitoneally injected with 0.5 ml NS. HPX (Abcam-ab198629, UK) was dissolved in 0.1% sodium azide and diluted to 1 μg/μl. Rats in the Heme + HPX group were intracerebroventricularly injected with 5 μl (5 μg) HPX. Rats in the control group and Heme group were intracerebroventricularly injected with 5 μl 0.1% sodium azide. The dose was selected based on previous studies [21, 30].

### Intracerebroventricular injection

Rats were anesthetized with isoflurane (2–3% isoflurane in O_2_ at 2 L/min). Anesthetized rats were placed on a stereotaxic apparatus and the skull was fixed and secured with four stainless-steel screws. An incision to the scalp exposed the surface of the skull and bregma. A burr hole was drilled into the bone of the right hemisphere with a stainless steel 26-gauge cannula, located 1.5 mm lateral to, and 0.8 mm posterior to the bregma. A 25 μl Hamilton micro syringe was very slowly inserted to 3.5 mm beneath the dural surface to inject 5 μl HPX or vehicle (0.1% sodium azide). The micro syringe was kept in place for 5 min to ensure the effectiveness of injection before withdrawal. After withdrawal, the incision was sutured, and the rats recovered from anesthesia.

### Behavioral tests

After injection of hemin or hemin + HPX, the rats were subjected to the MWM test to measure cognitive function (spatial learning and memory). The MWM test had two sessions: the acquisition session for five consecutive days and the probe trial session on day 6. In the MWM test, a black circular tank (150 cm in diameter and 50 cm in height) filled with water (22 ± 2 °C) was divided into four quadrants. A hidden platform (diameter 10 cm) was placed in the middle of one quadrant 1–1.5 cm below the water. The swimming path of each rat was recorded by video camera mounted directly above the tank. In the acquisition session, the rats were placed into the water facing the wall of the pool in one of the four quadrants. Each rat was allowed 120 s to find and mount the platform. When the rat found the platform, it was kept on the platform for 10 s. If the rat did not find the platform within 120 s, it was guided to the platform and allowed to stay on it for 10 s, and then the escape latency was recorded as 120 s. The escape latency, path length and swimming speed were recorded. In the probe trial session, 24 h after the last trial of the acquisition session, the original platform was removed. Rats were placed in the quadrant opposite to the platform quadrant and allowed to swim for 120 s. The number of platform crossings and time spent in the targeted quadrant were recorded.

### Cell culture and drug treatment

The BV2 mouse microglia cell line (FH0355) and HT22 mouse hippocampal neuron cell line (FH1027) was both purchased from Shanghai FuHeng Biology Co., Ltd. in China. On the first day, BV2 cells were maintained in Dulbecco’s modified Eagle’s medium (DMEM) (Gibco) supplemented with 10% fetal bovine serum (FBS) (Sigma–Aldrich, MO, USA) and 1% penicillin/streptomycin (Gibco) in humidified 5% CO_2_ air environment at 37 °C. On the second day, the BV2 cell culture medium was replaced, and the cells were divided into 4 groups: 1) the Control group (BV2), in which BV2 cells were incubated with medium only for 24 h; 2) the Heme group (BV2 + Heme) in which BV2 cells were treated with 40 μM hemin (H9039, Sigma, USA) for 24 h; 3) the MyD88 inhibition group (BV2 + Heme + T6167923) in which BV2 cells were treated with 40 μM hemin and 2 μM MyD88 inhibitor (T6167923, #HY-19744, MedChemExpress, USA) for 24 h; and 4) the NF-κB inhibition group (BV2 + Heme + JSH-23), in which BV2 cells were treated with 40 μM hemin and 20 μM NF-κB inhibitor (JSH-23, #S7351, Selleck, USA) for 24 h. Then, the BV2 cells were harvested for subsequent experiments.

### Transwell assay

The BV2 and HT22 cell coculture system was conducted using Corning Transwell polycarbonate membrane cell inserts (Corning Costar Corp, USA). BV2 cells were placed above the HT22 cell layer. HT22 cells were cultured in 24-well plates, and BV2 cells were seeded onto Transwell inserts. Coculture systems were divided into 4 groups: 1) Neuron group (HT22) in which only HT22 cells were incubated with medium for 24 h; 2) Neuron Heme group (HT22 + Heme) in which HT22 cells were treated with 40 μM hemin (H9039, Sigma, USA) for 24 h; 3) Neuron + microglia group (HT22 + BV2) in which HT22 cells cocultured with BV2 cells were incubated with medium for 24 h; 4) Neuron + microglia Heme group (HT22 + BV2 + Heme) in which HT22 cells cocultured with BV2 cells were treated with 40 μM hemin (H9039, Sigma, USA) for 24 h (Fig. 4A). Then, the coculture systems were harvested for subsequent experiments.

### Flow cytometry (FCM)

The complex of free heme and HPX could be recognized and cleared by CD91. Therefore, in the rat experiment, we detected the expression of CD91 in brain tissue by FCM. The brain tissue was dissected and minced into small pieces in 0.125% trypsin and 0.02% EDTA for digestion at 37 °C for 25 min. Then, brain tissue suspensions were passed through cell strainer (70 μm, Fisher brand) followed by incubation in culture bottles treated with poly L-lysine in 5% CO_2_ at 37 °C for 30 min. When only a few cells adhered to the wall, the cell suspension was centrifuged at 300 g at 4 °C for 5 min and resuspended in medium for counting. After washing with PBS, the cells were resuspended in binding buffer at a density of 1 × 10^6^ cells/ml and subjected to be incubated with a primary antibody (ab92544, Abcam) and then a secondary fluorescent antibody (ab7010, Abcam) in the dark at 4 °C for 30 min. Stained cells were spined down to a pellet and resuspended in the wash buffer for further analysis. In the cell culture experiment, HT22 cell apoptosis was assessed by Annexin V-FITC and propidium iodide (PI) (40302ES20, Yeasen Biotechnology, Shanghai, China). HT22 cells were digested with trypsin and centrifuged at 300 g at 4 °C for 5 min to wash. Cell pellets were resuspended in binding buffer at a density of 1 × 10^6^ cells/ml, and Annexin-FITC and PI were added. After reaction at room temperature for 15 min in the dark, binding buffer was added, and apoptotic cells were analyzed. Cells were analyzed on flow cytometer (FC 500, Beckman Coulter) as soon as possible, and the data were analyzed by FlowJo software (Informer Technologies, USA).

### Enzyme-linked immunosorbent assay (ELISA)

In rat experiment, the dorsal hippocampus was dissected based on the atlas of Paxinos and Watson and homogenized in RIPA lysis buffer followed by centrifugation at 13,000 × g for 10 min at 4 °C. The supernatant was collected, and the protein concentration was determined using bicinchoninic acid (BCA) assay kit (Beyotime Biotechnology, Shanghai, China). Commercially available ELISA kits for measuring TNF-α (CER1393, CRK Pharma, Wuhan, China), IL-1β (CER1094, CRK Pharma, Wuhan, China) and IL-6 (CER0042, CRK Pharma, Wuhan, China) levels were used according to the manufacturers’ instructions. In cell experiment, the culture medium of each group was collected, and the quantities of TNF-α (EK282, MultiSciences, Hangzhou, China), IL-1β (EK201B, MultiSciences, Hangzhou, China), and IL-6 (EK206HS, MultiSciences, Hangzhou, China) were determined using ELISA kits according to the manufacturer’s instructions.

### Immunofluorescence analysis

The expression of ionized calcium binding adaptor molecule 1 (Iba-1) was detected in rat experiment. The rat brain was fixed and sectioned. Then the sections were dehydrated with a series of graded ethanol solutions, cleared with distilled water, deparaffinized by xylene, hydrated with gradient alcohol and boiled for antigen retrieval. After blocking nonspecific binding with 3% bovine serum albumin (BSA)/10% normal goat serum for 30 min, the sections were incubated with primary anti-Iba-1 antibody (ab178846, Abcam) at 4 °C overnight. Then, the sections were washed and incubated with the secondary antibody goat anti-rabbit IgG-H&L (Alexa Fluor® 647) (ab150079, 1:1000, Abcam) at room temperature in the dark with slow shaking for 1 h. In the cell coculture experiment, HT22 cells were fixed, permeabilized, blocked, and incubated with primary antibody against neuron-specific nuclear protein (NeuN) (ab104224, Abcam) at 4 °C overnight. After washing, the cells were incubated with goat anti-mouse IgG-H&L (Alexa Fluor®647) (ab150115, 1:1000, Abcam) secondary antibody at room temperature in the dark with slow shaking for 1 h. In the BV2 cell culture experiment, the expression of CD86 was detected. The cells were fixed, permeated, blocked, and incubated with primary antibodies against CD86 (A16805, ABclonal) at 4 °C overnight. After washing, the cells were incubated with secondary antibodies at room temperature in the dark with slow shaking for 1 h. The nuclei were stained with 4′,6-diamidino-2-phenylindole (DAPI) and are shown in blue. Images were obtained by laser scanning confocal microscopy (Nikon, Tokyo, Japan), and ImageJ software (Bethesda MD, USA) was used for analysis.

### Western blot analysis

Western blot analysis was performed to detect the possible mechanism of the TLR4/MyD88/NF-κB signaling pathway in neuroinflammation induced by free heme in rat and BV2 cell experiments. Brain tissue from the rats or harvested BV2 cell pellets were homogenized in ice-cold RIPA lysis buffer (Beyotime Biotechnology) with the addition of protease and phosphatase inhibitor cocktail (Beyotime Biotechnology) to obtain total protein. Protein concentrations were quantified by BCA protein assay kit (Beyotime Biotechnology) according to the manufacturer’s instructions. Protein samples were suspended in loading buffer, boiled at 100 °C for 10 min, separated by 8–12% SDS-PAGE and transferred to polyvinylidene fluoride membranes (Millipore). The membranes were blocked with 5% skim milk for 1 h at room temperature and then incubated with primary antibodies at 4 °C overnight. In rat experiment, primary antibodies were as follows: anti-TLR4 (A5258, 1:1000, ABclonal, Wuhan, China); anti-MyD88 (ab219413, 1:1000, Abcam, Cambridge, UK); anti-p-NF-κB p-p65 (#3033, 1:1000, Cell Signaling Technology, Beverly, MA, USA); and anti-NF-κB p65 (#8242, 1:1000, Cell Signaling Technology, Beverly, MA, USA). In BV2 cell experiment, the primary antibodies were as follows: anti-TLR4 (19,811–1-AP, 1:1000, Proteintech, Wuhan, China); anti-MyD88 (ab219413, 1:1000, Abcam, Cambridge, UK); anti-p-NF-κB p-p65 (#3033, 1:1000, Cell Signaling Technology, Beverly, MA, USA); and anti-NF-κB p65 (10745–1-AP, 1:1000, Proteintech, Wuhan, China). The membranes were incubated with species-appropriate horseradish peroxidase (HRP)-conjugated secondary antibodies (1:5000, Cell Signaling Technology) for 1 h at 37 °C. Finally, the protein bands were visualized with enhanced chemiluminescence and quantified using ImageJ software.

### Statistical analysis

Statistical analysis in this study was conducted using SPSS 22.0 software (IBM Corp., Armonk, NY, USA) and GraphPad Prism 9 (GraphPad Software, San Diego, CA). One-way analysis of variance (ANOVA), two-way ANOVA, repeated ANOVA, and unpaired or paired Student’s t test were used to evaluate the differences among the groups, followed by Tukey’s post hoc test or Bonferroni’s post hoc test, as appropriate. Data are presented as means ± SEM. A value of *p* < 0.05 was set as statistical significance.

## Results

### Intraperitoneal injection of hemin induced learning and memory impairment

The MWM test was conducted to measure the cognitive function (spatial learning and memory) of rats. The swimming path of each rat was recorded (Fig. 1 A, B). In the acquisition session, the control group rats required the shortest time, and the Heme group rats required the longest time to find the target platform from day 1 to day 5. Rats in the control group and Heme + HPX group required less time on day 5 than on day 1 to find the target platform. While in the Heme group, the escape latency was not significantly different between day 5 and day 1. As the training progressed, the rats in the control group exhibited a discernible drop in escape latency. However, the Heme group did not show the same training performance as the control group (Fig. 1 C). No motor dysfunction was detected according to the swimming speed results (Additional file 1: Fig. S1). In the probe trial session, rats in the Heme group and Heme + HPX group crossed the platform less frequently and spent less time (%) in the target quadrant than those in the control group. Compared with the Heme group, the Heme + HPX group rats crossed the platform more frequently and spent more time (%) in the target quadrant (Fig. 1 B, D, E). The MWM test data indicated that Heme had negative effects on the spatial learning and memory abilities of rats and HPX could alleviate these effects.

### Hemin increased CD91 + cells and HPX eliminated excessive free heme in rat brain

HPX has high heme affinity, and heme-HPX complexes can be endocytosed by microglia. HPX has neuroprotective effect of keeping free heme at safe levels by preventing unregulated heme uptake [31]. CD91 is receptor protein for heme-HPX complexes. The uptake by CD91 leads to endocytosis and lysosomal degradation of the heme-HPX complexes [32]. So, the number of CD91 + cells represented the elimination condition of free heme. We hypothesized that free heme could enter the brain and activate the heme-HPX-CD91 scavenging system. FCM was performed to analyze the CD91 + cells and the results indicated that on day 7 after administration, CD91 + cells were significantly increased in the Heme group compared with the control group, and the Heme + HPX group had fewer CD91 + cells compared to the Heme group (Fig. 1 F, G, H). CD91 is the receptor to identify the heme-HPX complex. Therefore, the increase of CD91 + cells induced by hemin suggests that, there are still many heme-HPX complexes to be scavenged after a week of hemin administration. In addition, the decrease of CD91 + cells in the Heme + HPX group suggests that, HPX may help the brain clean excessive free heme, and the heme-HPX complexes have been cleaned faster compared to the Heme group within 7 days.

### Hemin induced neuroinflammation and HPX reduced neuroinflammation in rat brain

**Fig. 2:**
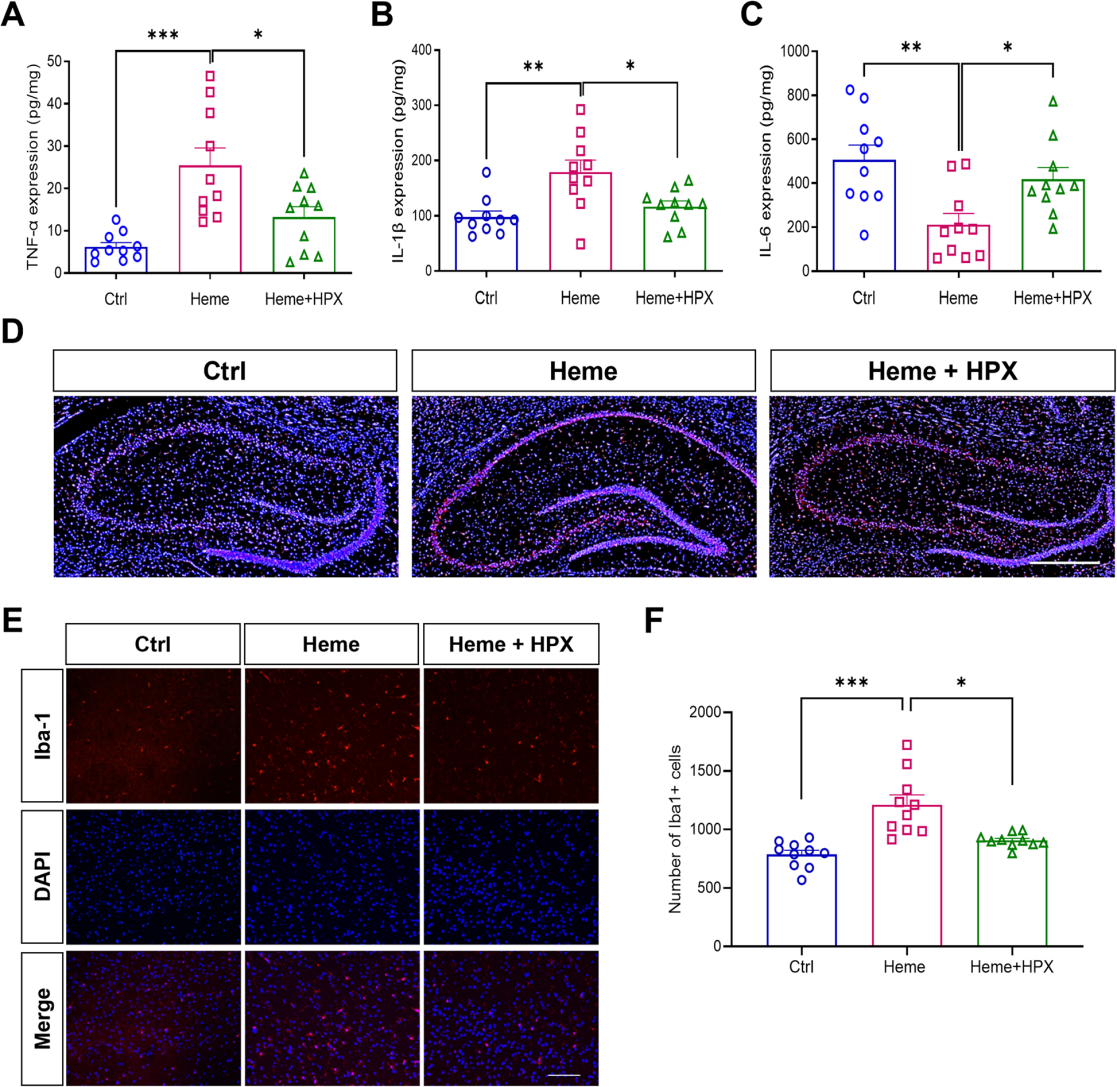
Hemin induced neuroinflammation and HPX reduced neuroinflammation in rat brain. Brain tissue ELISA analysis of TNF-α **A**, IL-1β **B** and IL-6 **C** on day 7 after administration in rats. **D** Representative immunofluorescence images of nuclei (blue) and Iba-1 + (red) staining in sections of hippocampus on Day 7 after administration. Scale bar = 500 μm. **E** Representative immunofluorescence images of nuclei (blue) and Iba-1 + (red) staining in brain cells on Day 7 after administration. Scale bar = 100 μm. **F** Quantitative analysis of Iba1 + cells in rat brain. Data are presented as means ± SEM. ***p < 0.001. **p < 0.01. *p < 0.05

To explore inflammation in rat brain, we took brain homogenates and detected the inflammatory cytokines TNF-α, IL-1β and IL-6. The ELISA analysis showed that, compared with the control group, TNF-α expression was significantly increased in the Heme group. Meanwhile, compared with the Heme group, TNF-α expression was decreased in the Heme + HPX group, suggesting that heme could induce inflammatory reaction in rat brain and that HPX treatment could reduce the reaction (Fig. 2A). Similarly, IL-1β expression was significantly increased in the Heme group compared with the control group and decreased in the Heme + HPX group compared with the Heme group (Fig. 2B). However, IL-6 expression was significantly decreased in the Heme group compared with the control group and increased in the Heme + HPX group compared with the Heme group (Fig. 2C), which might be related to the time of obtaining brain tissue, which was one week after administration.

To investigate the microglial response to free heme and HPX, rat brains were dissected, and immunofluorescence staining was used to assess the expression of Iba-1, which is a calcium binding protein specifically expressed in microglia. The results showed that Iba-1 was widely expressed in the hippocampus of rat brain (Fig. 2 D). We counted the number of Iba-1 + cells and found that compared with the control group, Iba-1 + cells were significantly increased in the Heme group. Meanwhile, the number of Iba-1 + cells was decreased in the Heme + HPX group compared with the Heme group (Fig. 2 E, F), suggesting that free heme could increase the number of microglia in rat brain and that HPX treatment could reduce the number of microglia. These findings indicate that free heme could induce activation of microglia and neuroinflammation in rat brain, but HPX treatment could reduce these effects.

### Hemin induced microglial inflammation and neuronal apoptosis

**Fig. 3:**
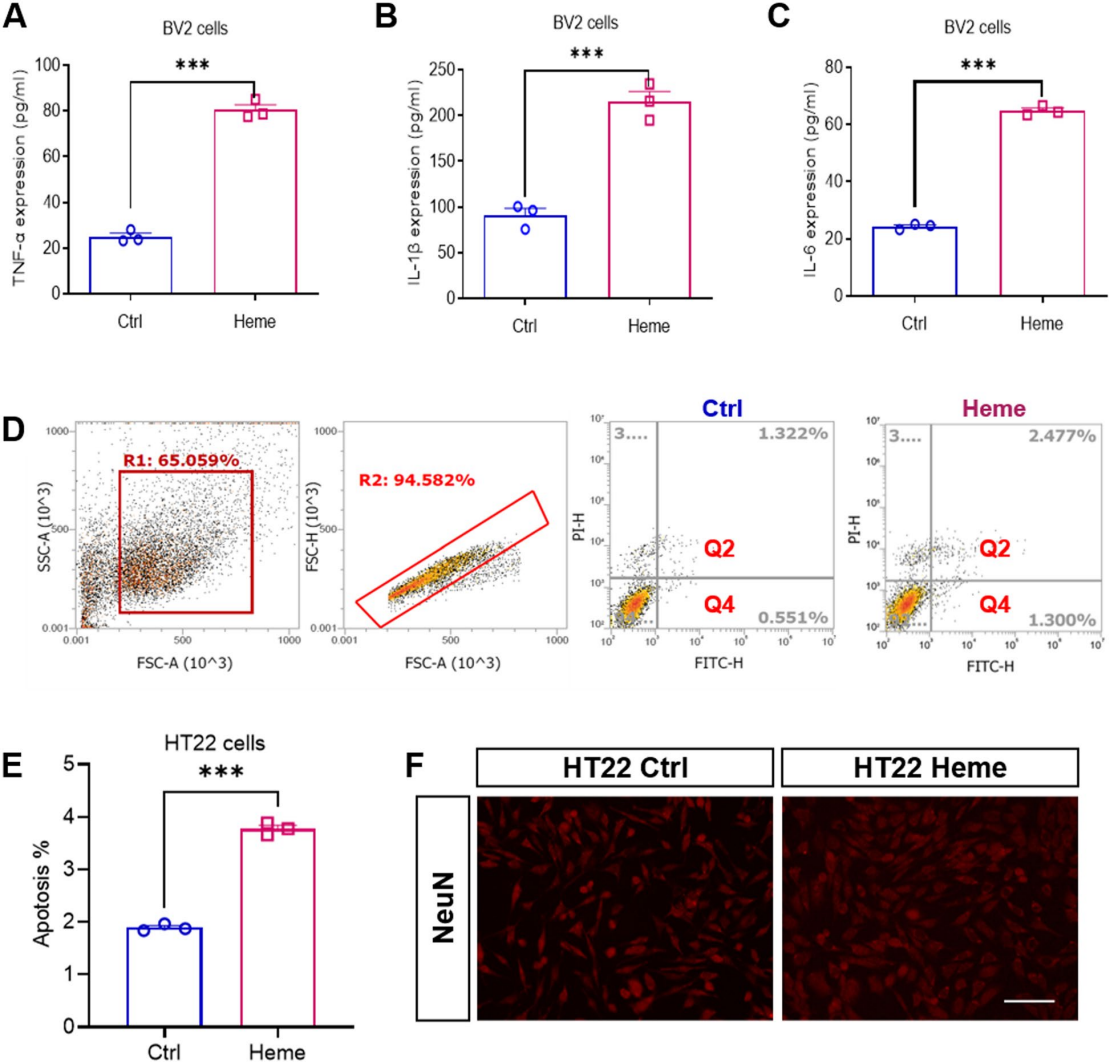
Hemin induced microglial inflammation and neuronal apoptosis. ELISA analysis of TNF-α **A**, IL-1β **B** and IL-6 **C** in BV2 cells after hemin treatment for 24 h. **D** The gating strategy and representative FACS plots of apoptotic cells (Q2 + Q4) in the control group and Heme group of HT22 cells. **E** Quantification of the percentage of apoptotic cells among HT22 cells in the control group and Heme group of HT22 cells. **F** Representative immunofluorescence images of neurons (red) in the control group and Heme group of HT22 cells. Scale bar = 100 μm. Data are presented as means ± SEM. ***p < 0.001

Microglia play an important role in the process of CNS injury and are the main effector cells of neuroinflammation. To investigate the inflammatory response of microglia induced by free heme, we conducted BV2 cell culture in vitro. After hemin treatment, we detected inflammation-related cytokines (TNF-α, IL-1β, and IL-6). ELISA analysis showed that compared with the control group, TNF-α, IL-1β and IL-6 expression was significantly increased in the Heme group of BV2 cells (Fig. 3 A, B, C). Next, FCM was conducted to determine whether hemin could induce neuronal apoptosis in HT22 cells. We found that the percentage of apoptotic cells was significantly increased in the Heme group compared with the control group (Fig. 3 D, E). Additionally, immunofluorescence staining was conducted to observe neurons (Fig. 3 F). These findings indicated that hemin significantly induced microglial inflammation and neuronal apoptosis.

### Microglia aggravated free heme-induced neuronal apoptosis

**Fig. 4:**
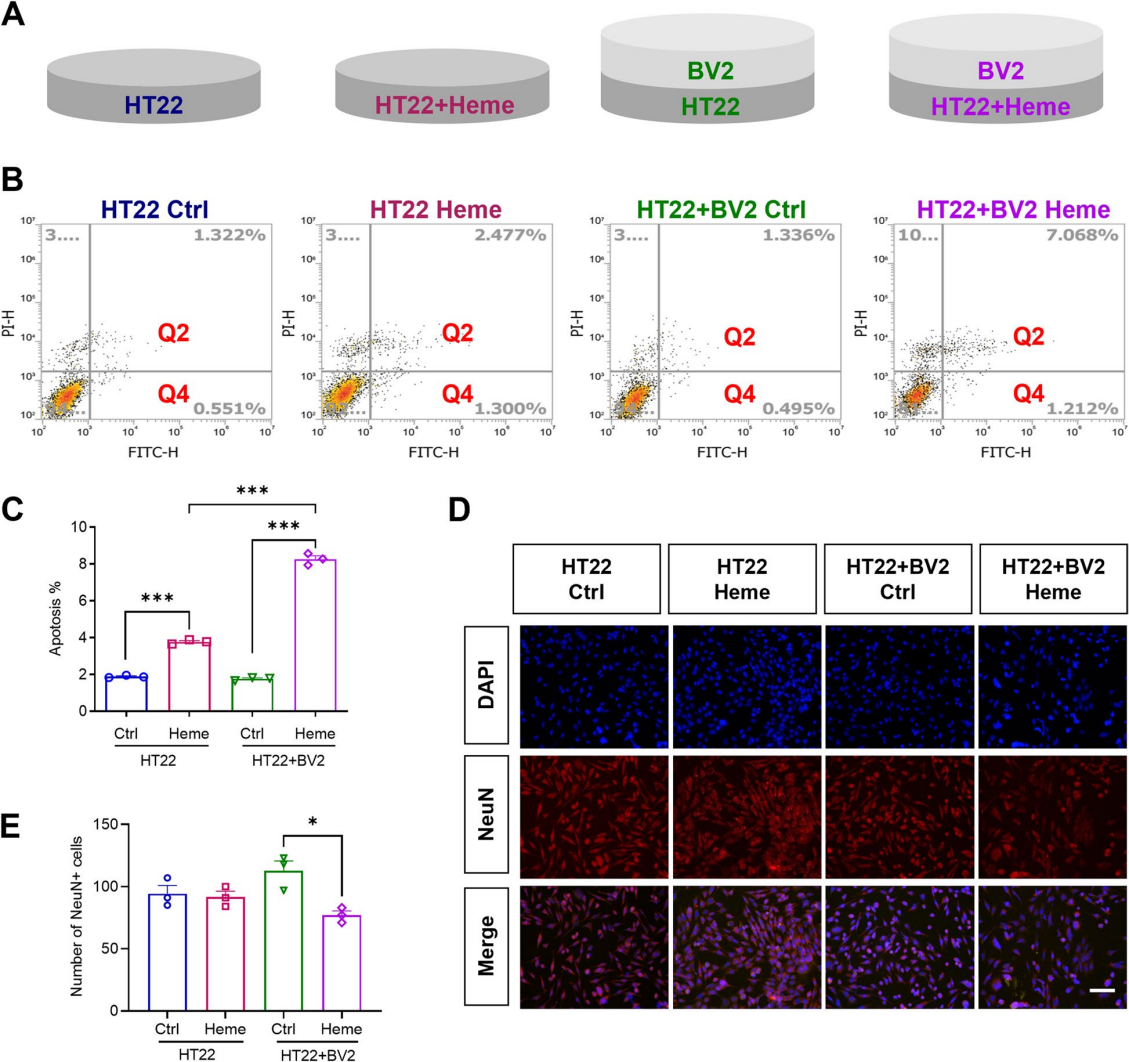
Microglia aggravated free heme-induced neuronal apoptosis.** A** BV2 cell and HT22 cell coculture system and four groups: 1) Neuron group (HT22) in which only HT22 cells were incubated for 24 h; 2) Neuron Heme group (HT22 + Heme) in which HT22 cells were treated with hemin for 24 h; 3) Neuron + microglia group (HT22 + BV2) in which HT22 cells cocultured with BV2 cells were incubated for 24 h; 4) Neuron + microglia Heme group (HT22 + BV2 + Heme) in which HT22 cells cocultured with BV2 cells were treated with hemin for 24 h.** B** Representative FACS plots of apoptotic cells (Q2 + Q4) in four groups. **C** Quantification of the percentage of apoptotic cells among HT22 cells in four groups. **D** Representative immunofluorescence images of nuclei (blue) and NeuN (red) staining among HT22 cells in four groups. Scale bar = 100 μm. **E** Quantitative analysis of NeuN + cells in four groups. Data are presented as means ± SEM. ***p < 0.001. *p < 0.05

To verify that microglia were involved in neuronal apoptosis, we cocultured BV2 cells and HT22 cells with hemin treatment (Fig. 4 A) and detected neuronal apoptosis in HT22 cells by FCM. We found that in HT22 cells cultured alone or in the HT22 + BV2 cell coculture system, hemin significantly induced neuronal apoptosis. Notably, compared with that in the HT22 Heme group, the apoptosis of neurons in the HT22 + BV2 Heme group was significantly increased, indicating that microglia were involved in and increased neuronal apoptosis (Fig. 4 B, C). Then, immunofluorescence staining was used to observe HT22 cells in four groups (Fig. 4 D). NeuN antibodies were tools for staining live mature neurons. In HT22 cells alone, hemin didn’t reduce the number of NeuN + cells. While in the coculture system, hemin significantly reduced the number of NeuN + cells (Fig. 4 E). Therefore, the results suggests that microglia aggravated free heme-induced neuronal apoptosis. Furthermore, hemin might decrease the number of live mature neurons under the effect of microglia.

### TLR4/MyD88/NF-κB signaling pathway participated in free heme-induced neuroinflammation and cognitive dysfunction

**Fig. 5:**
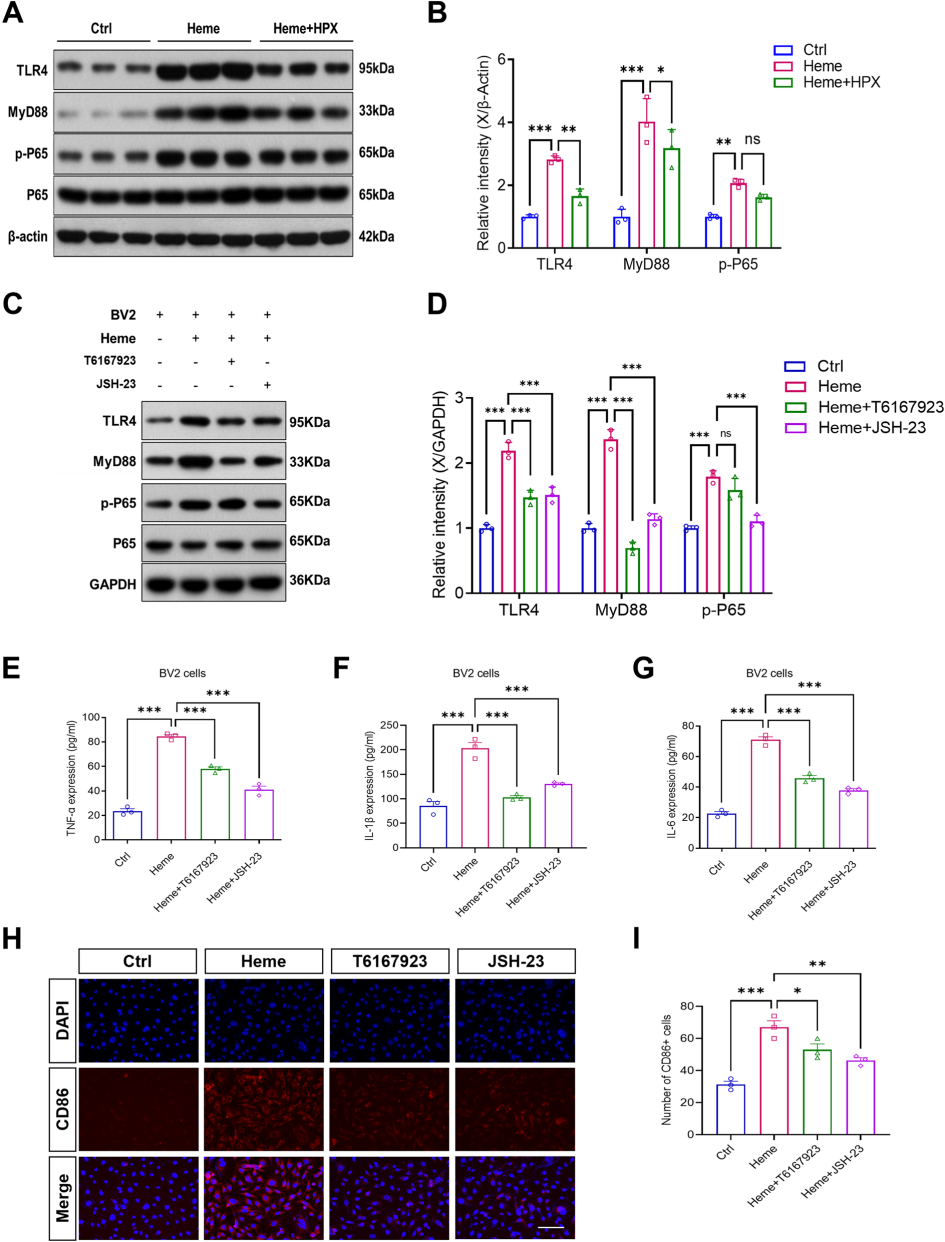
TLR4/MyD88/NF-κB signaling pathway participated in free heme-induced neuroinflammation and cognitive dysfunction. **A** Representative western blot bands of TLR4, MyD88, p-P65 and P65 in rat brain tissue on day 7 after administration. **B** Relative intensity of TLR4, MyD88 and p-P65 protein expression in rat brain tissue on day 7 after administration. **C** Representative western blot bands of TLR4, MyD88, p-P65 and P65 in BV2 cells after treatment for 24 h. BV2 cells were divided into 4 groups: 1) the Control group; 2) the Heme group treated with hemin; 3) the Heme + T6167923 group treated with hemin and MyD88 inhibitor; and 4) the Heme + JSH-23 group treated with hemin and NF-κB inhibitor. **D** Relative intensity of TLR4, MyD88 and P65 in BV2 cells after treatment for 24 h. ELISA analysis of TNF-α **E**, IL-1β **F** and IL-6 **G** in BV2 cells after treatment for 24 h. **H** Representative immunofluorescence images of CD86 (red) staining in BV2 cells. Scale bar = 100 μm. **I** Quantitative analysis of CD86 + cells. Data are presented as means ± SEM. ***p < 0.001. **p < 0.01. *p < 0.05. ns *p* > 0.05

We assumed that free heme could cause microglial inflammation and neurocognitive dysfunction by regulating the TLR4/MyD88/NF-κB signaling pathway. In the rat experiment, to verify this hypothesis, we detected TLR4, MyD88, p-P65 (p-NF-κB) and P65 (NF-κB) proteins in rat brain tissue by western blot (Fig. 5 A). The results indicated that, compared with the control group, hemin significantly increased the protein levels of TLR4, MyD88 and p-P65. Notably, compared with Heme group, HPX significantly reduced the protein levels of TLR4 and MyD88 (Fig. 5 B).

In cell culture experiment, to further evaluate the effects of hemin on the regulation of the pathway, BV2 cells were treated with hemin or hemin + inhibitor for 24 h. Western blot analysis results revealed that hemin significantly increased TLR4, MyD88 and p-P65 protein levels compared with the control group. As expected, the MyD88 inhibitor (T6167923) and NF-κB inhibitor (JSH-23) significantly downregulated hemin-induced pathway protein levels. Interestingly, the protein level of TLR4 was decreased by MyD88 inhibitor (T6167923) and NF-κB inhibitor (JSH-23) treatment compared with that in the Heme group, which might be related to the positive feedback regulation of the pathway (Fig. 5 C, D). Next, we hypothesized that inhibiting the pathway could reduce the inflammatory response of microglia. To verify this hypothesis, we detected inflammatory cytokines. ELISA analysis showed that the MyD88 inhibitor (T6167923) and NF-κB inhibitor (JSH-23) significantly decreased hemin-induced TNF-α, IL-1β and IL-6 expression in BV2 cells (Fig. 5 E, F, G).

Then, we observed the expression of the M1 surface marker CD86 in BV2 cells. Consistent with the inflammatory cytokine ELISA results, the CD86 + cells were significantly increased in the Heme group compared with the control group, and MyD88/NF-κB inhibition reversed the increase of CD86 + cells compared to the Heme group (Fig. 5 H, I). Overall, the data demonstrated that free heme could cause neuroinflammation by microglial activation via the TLR4/MyD88/NF-κB signaling pathway.

## Discussion

**Fig. 6:**
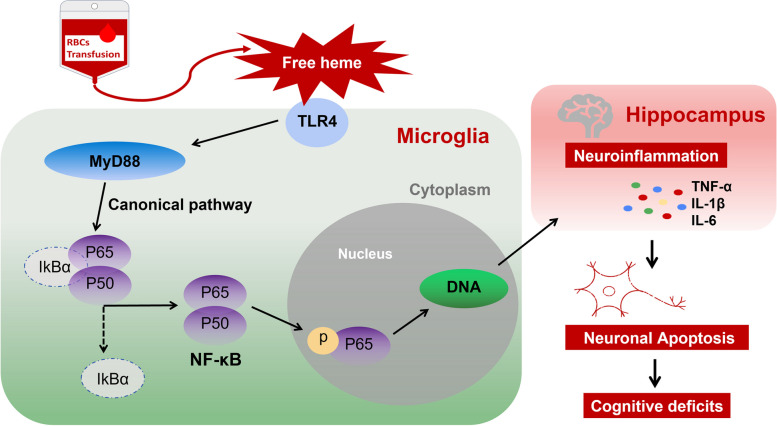
General overview of the molecular mechanisms of PND after RBCs transfusion. Free heme induces neuroinflammation and cognitive impairment by microglial activation via the TLR4/MyD88/NF-κB signaling pathway

Intraoperative blood transfusion is one of the consistent risk factors for PND across multiple surgical specialties [16]. Our study is the first to focus on the mechanism of neuroinflammation and cognitive impairment caused by free heme after RBCs transfusion. We provided several lines of evidence supporting the hypothesis that free heme could induce neuroinflammation and cognitive deficits by microglial activation via the TLR4/MyD88/NF-κB signaling pathway (Fig. 6).

The MWM is commonly used to test hippocampus-dependent learning and spatial memory [33]. In this study, by behavioral tests of MWM in rats, we showed that hemin induced learning and memory impairment and that HPX could reduce it. Heme is synthesized in all nucleated cells, and cellular levels are controlled by enzymatic synthesis and degradation. It has contradictory biological functions as a physiological and toxic molecule [34]. First, heme is essential for oxygen transport and storage in Hb. On the other hand, free heme can cause oxidative stress and plays an important role in the pathogenesis of neurological diseases [35]. However, the mechanism of free heme -induced PND after RBCs transfusion is less clear.

Evidence has shown that for patients who received RBCs transfusion, the levels of free heme in peripheral blood were dependent on the number of RBCs units transfused [12]. Therefore, we could not find direct evidence to show the lowest free heme level that could cause neuroinflammation or cognitive deficits. We chose 50 mg/kg hemin for a single intraperitoneal injection in rat model based on previous studies [27, 29]. However, heme is a double-edged sword with contradictory effects. It is vital cellular component of Hb, but excessive levels of free heme have pro-oxidant and proinflammatory effects. In previous studies, the results of hemin treatment in different disease models by intraperitoneal or intracerebral injection might be inconsistent. In some studies, pretreatment with small-dose hemin upregulated HO-1 levels, which had an antioxidant effect, and the results showed neuroprotective effect in which the induction of HO-1 could alleviate neuronal damage [26]. In contrast, in other studies, such as intracerebral injection of hemin or Hb or autologous blood in cerebral hemorrhage model, the results showed hemin-induced neuroinflammation and neurological deficits [20, 36]. Different from studies about cerebral ischemia or cerebral hemorrhage, we injected hemin into healthy rats, and our data demonstrated a potential neurotoxic role of free heme-induced neuroinflammation and cognitive deficits. Based on the results, we speculated that the neurotoxicity of free heme was dose dependent, which might be consistent with the evidence of clinical studies on RBCs transfusion that the higher the level of free heme after RBCs transfusion is, the more serious the cognitive abnormality [7, 8]. Therefore, we further speculated that the severity of cognitive impairment after RBCs transfusion was related to the amount of transfusion, length of RBCs storage time and the patients’ hemolytic condition.

HPX is a 60-kDa acute-phase binding protein with high heme affinity. HPX in brain tissue has two sources: accumulation from an intrathecal origin and transfer from the circulation. Heme-HPX complexes are endocytosed by receptor-mediated mechanism in a variety of cells, including hepatocytes, macrophages, and syncytiotrophoblasts [21, 22]. We chose the dose of HPX (5 μg, 5 μl) for intraventricular injection in the rat model based on previous study [30]. Our results were consistent with previous studies that reported HPX could alleviate cognitive dysfunction after focal cerebral ischemia–reperfusion injury in rats [37]. As a receptor protein for heme-HPX complexes, CD91 could expressed in neurons and glia of brain tissue but not expressed by cerebral endothelium [22]. So, the CD91 + cells in our study might be from neurons and glia, but not other cells or debris. After one week of hemin administration, there were still more CD91 + cells in rat brain, indicating that the clearance process is long lasting. This finding also suggests that HPX plays a potential role in both early and late treatment.

Microglia are known as resident innate immune cells in the CNS and play a central role in PND. Iba-1 is a histological microglial marker, and our data showed that free heme could increase the number of Iba-1 + cells in the hippocampus of rat brain. However, we did not perform morphological analysis of microglia. There is a particular balance between proinflammatory and anti-inflammatory cytokines released by microglia. The levels of TNF-α, IL-1β and IL-6 are frequently used to evaluate inflammation. Furthermore, we revealed free heme-induced increase in TNF-α and IL-1β and reduction in IL-6 expression on day 7 after hemin administration. Our findings regarding TNF-α and IL-1β are consistent with numerous previous studies on the pathophysiology of neuroinflammation showing that increased levels of proinflammatory cytokines TNF-α and IL-1β are related to neuronal cytotoxicity [38].

The production of IL-6 is upregulated in many neuroinflammatory diseases [39]. It has been reported that transfusion of old RBCs or intravenous injection of free-Hb could increase IL-1β and IL-6 in the cerebral cortex and hippocampus of rats at 24 h [9], which was consistent with our results that in BV2 cells, hemin increased inflammatory cytokines at 24 h. However, the change in IL-6 levels in the brain at 7 d after RBCs transfusion has not been studied before. We speculate that the reasons for the IL-6 decrease on day 7 involve three aspects: 1) In the pathological process of different diseases, the level and distribution of microglial markers will change dynamically over time [40]. Recently, a study found that changes in plasma IL-6 can predict changes in cognitive function one year after surgery [41]. Therefore, the results at 24 h and day 7 may be different. 2) The induction of each inflammatory cytokine advances via a different signaling pathway in microglia [38], so the results for IL-6 levels in rat brain are different. 3) IL-6 has been shown to have both beneficial and destructive actions, triggering either neuronal survival after injury or causing neuronal apoptosis [42], so IL-6 may play a potential role in nerve repair in rats on day 7 after hemin administration. In conclusion, in rat experiment, our findings showed free heme-induced microglial activation in the hippocampus and cognitive deficits, and HPX had neuroprotective effect.

In our current study, the in vitro findings demonstrated increased expression levels of TNF-α, IL-1β and IL-6 in BV2 cells and increased apoptosis in HT22 cells separately treated with hemin after 24 h, suggesting free heme-induced neuroinflammation. Evidence has shown microglia-mediated neuronal cytotoxicity [43], which is consistent with our observation that free heme-induced neuronal apoptosis is dependent on the presence of microglia.

Finally, we show that the TLR4/MyD88/NF-κB signaling pathway may be involved in free heme-induced neuroinflammation and cognitive deficits. TLR4 is linked to various pathophysiological conditions and can be activated by numerous extraneous and endogenous molecules that are essential for neuroinflammatory responses [24]. Heme has been suggested to be a direct and indirect trigger of TLR4 activation [44]. MyD88 is a crucial downstream signaling ligand of TLR4 receptor and an essential adapter protein of the NF-κB signaling pathway. Phosphorylation of NF-κB regulates the transcriptional activity of NF-κB in the nucleus, triggering the production of inflammatory factors. Consistently, studies have proven that regulating the TLR4/MyD88/NF-κB pathway in microglia might take part in neuroinflammation and cognitive impairment [45, 46]. Microglia can be activated to the M1 phenotype by producing proinflammatory mediators or to the M2 phenotype by producing anti-inflammatory mediators [47]. We demonstrated that inhibiting the pathway could decrease the expression of inflammatory factors and CD86 which is commonly used as a marker for M1 microglia.

Some limitations should be noted in our study. First, the dosage of hemin used in rat and cell experiments was single, and we could not draw conclusions about the effects of free heme at lower or higher concentrations. Further studies on the effects of different levels of free heme on the CNS are necessary to clarify the role of RBCs transfusion in PND. Meanwhile, the dosage of HPX was also single, and the optimal HPX dose to scavenge free heme in the brain could not be determined. Second, HO-1 is an important heme-catabolizing enzyme, and its change in expression has been demonstrated in a variety of inflammatory conditions [23]. However, we did not conduct research on HO-1. Finally, we only examined the TLR4/MyD88/NF-κB signaling pathway, but several other pathways may be involved in the free heme-induced inflammatory response.

## Conclusion

In conclusion, we demonstrated that free heme could induce neuroinflammation and cognitive deficits in rats. Combined with in vitro experiments, we showed that the TLR4/MyD88/NF-κB signaling pathway may be involved in the pathophysiological mechanisms. Our findings may provide a better understanding of the relationship between intraoperative RBCs transfusion and PND. Chelating free heme or inhibiting neuroinflammation may become new therapeutic targets of PND for patients receiving RBCs transfusion.

### Supplementary Information

**Additional file 1: Supplementary Fig. S1 **The swimming speed **A **and path length in the target quadrant** B** in the acquisition session of MWM test.

## Data Availability

The datasets used and/or analysed during the current study are available from the corresponding author on reasonable request.

## Abbreviations

RBCs: Red blood cells
Hb: Hemoglobin
CNS: Central nervous system
POD: Postoperative delirium
PND: Perioperative neurocognitive disorders
POCD: Postoperative cognitive dysfunction
HPX: Hemopexin
HO-1: Heme oxygenase-1
TLR4: Toll-like receptors 4
MyD88: Myeloid differentiation factor 88
NF-κB: Nuclear factor-kappa B
TNF-α: Tumor necrosis factor-alpha
IL-1β: Interleukin-1β
IL-6: Interleukin-6
NS: Normal Saline
MWM: Morris water maze
FCM: Flow cytometry
ELISA: Enzyme-linked immunosorbent assay
Iba-1: Ionized calcium binding adaptor molecule 1
NeuN: Neuron-specific nuclear protein
