# Supplementary figures and images for: Free heme induces neuroinflammation and cognitive impairment by microglial activation via the TLR4/MyD88/NF-κB signaling pathway
Source: Cell Commun Signal. 2024 Jan 5;22:16. doi: 10.1186/s12964-023-01387-8 (PMC10768134; doi:10.1186/s12964-023-01387-8)

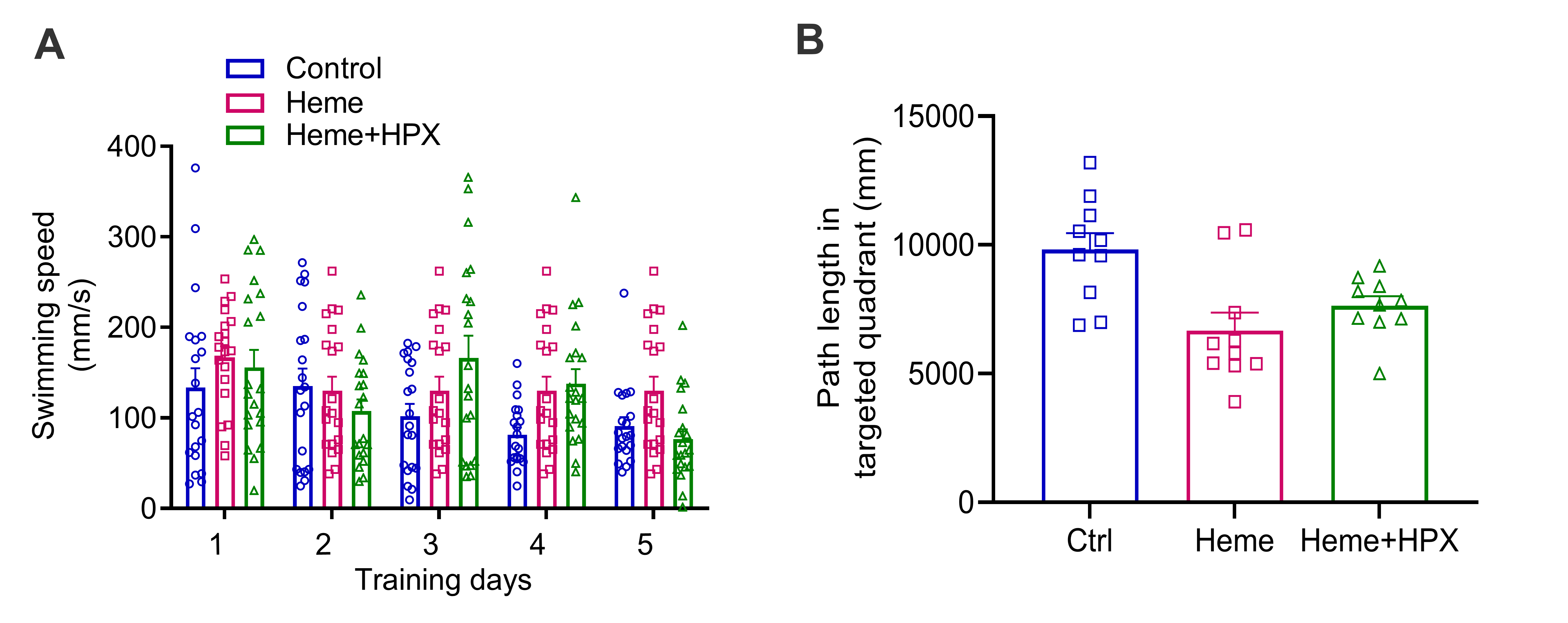

Supplement: Supplementary file 2 — Additional file 1: Supplementary Fig. S1 The swimming speed A and path length in the target quadrant B in the acquisition session of MWM test. [file 12964_2023_1387_MOESM1_ESM.tif]
